# Translation and cultural adaptation of the Integrated Palliative care Outcome Scale for Dementia (IPOS-Dem) to Swedish

**DOI:** 10.1186/s12912-022-00859-5

**Published:** 2022-04-02

**Authors:** Lisa Martinsson, Klas-Göran Sahlén

**Affiliations:** 1 Department of Radiation Sciences, Umeå University, 901 87 Umeå, Sweden; 2 Epidemiology and Global Health, Umeå University, 901 87 Umeå, Sweden

**Keywords:** Palliative care, Dementia, Outcome measurement, Qualitative research, Healthcare quality

## Abstract

**Introduction:**

Systematic assessment tools are helpful for improving and maintaining quality of care. The Integrated Palliative care Outcome Scale (IPOS) was developed for systematic assessment of persons receiving palliative care in a patient-centred way. A version of this tool, the Integrated Palliative care Outcome Scale for Dementia (IPOS-Dem), has been developed for patients with dementia.

The aim of this study was to develop a version of the IPOS-Dem translated into Swedish and culturally adapted to a Swedish care setting.

**Methods:**

Forward and backward translations from English into Swedish were performed to develop a first Swedish version. This version was modified for clarity and cultural adaptation based on 13 interviews with nurses and assistant nurses working in geriatrics and dementia care homes.

**Results:**

The interview process revealed several issues with the first version that emerged from the translation process. This was changed and further tested to work well, resulting in the final version of the Swedish IPOS-Dem. The tool was perceived as clinically useful but somewhat overlapping with already implemented tools for assessing behavioural and psychological symptoms in dementia.

**Conclusion:**

The Swedish version of the IPOS-Dem can now be used as a tool for assessing palliative care related problems and concerns for persons with advanced dementia. Future studies can focus on implementation as well as examining validity and reliability of this tool in a Swedish context.

## Background

Structured assessment instruments are commonly used in Sweden to support quality in healthcare and elderly care. These are often connected to a quality register, which collects data about care for individuals in specific topics. These data are then used for quality monitoring, research and sometimes resource allocation. One example of a quality register commonly used in Swedish elderly care is Senior alert. Senior alert is a quality register for adults 65 years and older. It focuses on risk assessments regarding nutrition, pressure ulcers, falls and oral health [1]. Another quality register commonly employed in Swedish elderly care is the BPSD (behavioural and psychological symptoms of dementia) registry. The BPSD registry focuses on non-pharmacological interventions to manage BPSD [2].

In Sweden, almost 1% (82,000 people) of the total population live in nursing homes and many residents are cared in their nursing home also during end of life. Cardiovascular diseases and dementia are very common diagnoses and the residents often have multiple diseases. Time from admission to death is two years in median but varies a lot between different regions [3].

Person-centred care for persons with dementia is a philosophy of care built around the needs of the individual [4]. It has been shown to increase quality of life and diminish neuropsychiatric symptoms compared to regular care [5]. Palliative care aims to optimise quality of care and diminish suffering for persons with life-threatening illnesses and for their families. In 1999, the Palliative care Outcome Scale (POS) was developed to assess symptoms and other problems for individuals receiving palliative care. Based on POS and POS-S (a symptom module), The Integrated Palliative care Outcome Scale (IPOS) was developed [6]. IPOS has been translated into several languages, including Chinese [7], Italian [8], Estonian [9], French [10], Czech [11], Turkish [12], Portuguese [13] and Japanese [14]. A Swedish version of IPOS has been developed by Beck et al. [15] and has now been partly implemented in specialised palliative care in Sweden.

The Integrated Palliative care Outcome Scale for Dementia (IPOS-Dem) was developed by Ellis-Smith et al. to accommodate the palliative care related problems and concerns for people with dementia in long-term residential care homes, since there was a need to assess additional symptoms and problems relevant to this population that were not covered in the IPOS [16]. Patient-reported measurements are generally regarded more accurate but is not always achievable. The IPOS-Dem is to be answered by healthcare professionals (proxy-reported), since it is to be used in a patient population that cannot answer a questionnaire themselves. The use of IPOS-Dem is recommended when a person moves into a care home and then regularly afterwards. The instrument consists of 28 symptoms and problems, and their severity is rated from zero to four [16]. A German version of the IPOS-Dem has been developed [17], and IPOS-Dem has also been tested for implementation in the United Kingdom in a residential care home setting. The introduction into standard care was found to be both feasible and acceptable [18].

### Aim

The aim of this study was to develop a version of the IPOS-Dem translated into Swedish and culturally adapted to a Swedish care setting. A secondary aim was to examine healthcare workers’ first impression on the usefulness of the instrument in a Swedish care context.

## Methods

### IPOS-Dem tool

The first question is an open question about the main problems the person with dementia has had over the past week. The second question concerns 19 individual symptoms/problems, with the option to write three more symptoms/problems, which are all scored with a 0–4 Likert scale (from “not at all” to “overwhelmingly”) or “cannot assess”. Questions 3, 4, 5a, 5b, 6, 7a, 7b and 8 evaluate psychological and social well-being and are scored with a 0–4 Likert scale (from “not at all” to “always”) or “cannot assess”. Question 9, about practical problems, is scored with a 0–4 Likert scale (from “problems addressed/no problems” to “problems not addressed”) or “cannot assess”. There are also questions soliciting background information about the person with dementia, including weight.

Permission to translate IPOS-Dem into Swedish was given by the developers at Cicely Saunders Institute, King’s College, London.

### Translation process

We based our translation process on guidelines for translation of the IPOS family instrument, available at the IPOS web page [19]. We also followed general guidelines for the translation and cultural adaptation process [20, 21].

Phase 1 was a brief literature review of the concepts. Also, the first author participated in an IPOS-Dem consolidation workshop hosted by the original developers, where concepts were discussed.

In phase 2, two translators with Swedish as their first language, one working in the healthcare field and one without healthcare knowledge, each translated the IPOS-Dem into Swedish. The two versions were then merged by the authors, and the merged version was discussed with the translators via e-mail. The Swedish version was then translated back into English by two translators with English as their first language, one with and one without a healthcare background. All versions of IPOS-Dem were then used to create a final translation of IPOS-Dem into Swedish. The final version was created by the authors in communication with the translators via e-mail.

The manual for IPOS-Dem was translated into Swedish by a professional translator.

### Interviews

**Fig. 1:**
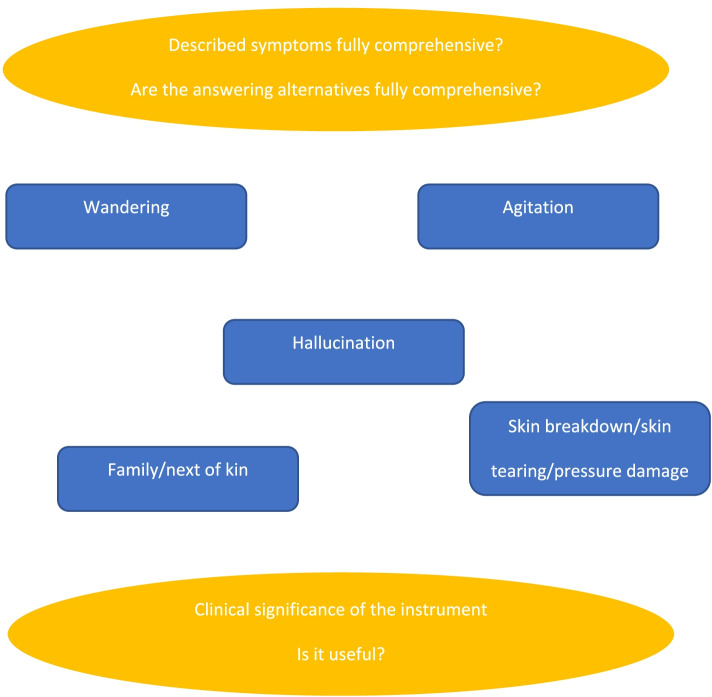
Interview guide for cognitive interviews about IPOS-Dem

During the interviews, the comprehension of all parts of the IPOS-Dem was addressed. The interviewed persons were asked to share their thoughts and uncertainties during the process. An interview guide was developed with an over-arching question about all included parts of the tool from the top to the bottom, and with special focus on the translation problems identified in the prior steps to be asked when these concerns were not addressed in the first phase of the interview; see Fig. 1. The implication of this was that the design of the interview guide emerged from the interviews conducted earlier, and thus was slightly changed. A problematic part of the suggested translation was discussed and a revision was produced. This revision was later discussed in the subsequent interviews and was either approved or disputed. Step by step, the translation was improved and accepted.

Managers for residential care homes and a geriatric hospital ward were contacted to recruit healthcare workers for individual interviews. That resulted in booking interviews with 15 persons, of whom one declined participation upon the meeting and one was excluded due to language barriers, and 13 interviews were conducted. The sampling procedure were purposive from the aspect that we wanted to reach experienced staff, but it can also be regarded as convenience sampling since we got informants by suggestion from managers. All interviewed persons had clinical experience of caring for people with advanced dementia. There was no relationship established prior to study commencement between the interviewer and the participants. Thus, prior to the start of the interview, the interviewer(s) presented themselves and the reason for conducting the research.

All interviewed persons received written and spoken information about the study and gave their written informed consent before the interview started. The first three interviews were conducted face-to-face (two at the interviewed persons’ workplaces and one at the first author’s workplace) and the last ten were conducted digitally via Zoom, due to the Covid-19 pandemic. The interviews lasted between 29 to 54 min each with a median of 40 min. The interviews were audio recorded and later transcribed. Both authors conducted interviews number 1, 2, 4, 6 and 13 together, and the first author, LM, conducted the other eight interviews. Field notes were made during the interviews. No one besides the interviewed person and the researcher(s) participated. The interviews were inspired by cognitive interview technique and our informants had the possibility to speak freely regarding all parts of the instrument. We also ended all interviews with a reflection upon the usefulness of the instrument and a summary of the content of the interview. The interview guide was not formally pilot tested, however since we used an emergent design the guide slightly changed over time. Transcripts were not returned to participants for comments or corrections, and the participants were not asked to provide feedback on the findings. Background information about the persons interviewed is given in Table 1.

**Table 1:** Background information about the interviewees (*n* = 13)

Occupation	Nurse	4
	Assistant nurse	9
Age (years)	Median	45
Sex	Women	13
Workplace	Geriatric department	3
	Elderly care facility	10

The analysis process was inspired by a theoretical thematic analysis [22]. Both authors started familiarising themselves with the data and continued to identify relevant ideas. The IPOS-Dem items guided the themes in focus during the interviews and the analysis process. After five interviews the translated version of IPOS-Dem was revised according to the findings in the first interviews. After the sixth interview, additional minor changes were made. To check for saturation, seven additional interviews were conducted but no additional information emerged, so we assessed that it was not probable that important new information would emerge in further interviews.

## Results

### Conceptual phase

In phase 1, we concluded that all main items included in the original IPOS-Dem are mentioned in either the Swedish national guidelines for palliative end-of-life care [23] or the dementia guidelines [24]. Skin tearing was identified early as the only term in IPOS-Dem without a self-evident Swedish equivalent. A literature search was made and a study was found that translated the International Skin Tear Advisory Panel (ISTAP) classification system for skin tears into Swedish. In that study, a survey among healthcare professionals was conducted to find the best possible match for “skin tear” in Swedish. The term “hudfliksskada” was identified [25]. Because the term had been newly suggested and was not fully established, we first decided to omit a specific term for skin tearing and test in the interviews whether skin tearing was perceived as being included in the wider term “skin breakdown”. To signal that the three terms given as examples in parentheses were just examples, we added “for example” before those words.

### Forward translation

The two forward translators suggested different wordings to explain “wandering”, but they both suggested the main term “vandringsbeteende” in Swedish. After discussion with the translators, the authors omitted the explanation, as the Swedish term for wandering was perceived as self-explanatory.

The translators also suggested different translations for the explanations in parentheses of the terms nausea and vomiting. As above, these explanations were omitted in the first merged Swedish version. It was noted that this was also done in the Swedish translated version of IPOS [15].

The two translators suggested different terms for the response endpoint “overwhelmingly”, namely, “mycket allvarligt” and “outhärdligt”. Neither of the translators suggested the direct translation “överväldigande”, which was perceived as a word with a too-positive connotation in Swedish to be used in this context. The authors decided to keep both variants in the backward translation to get more input into which wording to use.

Otherwise, the two Swedish translations were similar.

### Backward translation

The two Swedish translations for “overwhelmingly” were back-translated as “very seriously” and “unbearable”. The authors concluded that none of the Swedish words suggested in the forward translation was backward translated close to the original, suggesting the need for another solution. The authors for the Swedish translation of IPOS, Beck et al., had also noted this difficulty [15]. We decided to use the same terms as in commonly used pain assessment tools, the visual analogue scale (VAS) and the numeric rating scale (NRS) [26, 27].

The backward translation process did not suggest any other problems with the Swedish translation.

### Interviews

After five interviews, several changes were made to the instrument, see Table 2. The word “early” was added before the phrase “integrated palliative care” on the front page. That was because the interviewees expressed confusion as to which patients/residents the tool was intended for. The word “palliative” suggested supportive care during the last days of life for persons imminently dying. This change was tested and found to suggest palliative care in a broader perspective.

**Table 2:** All items in the English IPOS-Dem, the interview person’s comprehension of the item and whether the item was changed based on the interviews

Item in the English IPOS-Dem	Interview persons’ comprehension	Changes made to the Swedish translation based on the interviews
Q1. What have been the person’s main problems over the past week?	Good comprehension	No
Q2. Please select one box that best describes how the person has been affected by each of the following symptoms over the past week	Good comprehension	No
Pain	Good comprehension, but concerns raised about how to assess pain	No
Shortness of breath	Good comprehension	No
Weakness or lack of energy	Good comprehension	No
Nausea (feeling like being sick/vomiting)	Good comprehension without the explanation in parentheses	Yes
Vomiting (being sick)	Good comprehension without the explanation in parentheses	Yes
Poor appetite	Good comprehension	No
Constipation	Good comprehension	No
Dental problems or problems with dentures	Good comprehension	No
Sore or dry mouth	Good comprehension	No
Drowsiness (sleepiness)	The word in the parenthesis was better understood and more commonly used compared to the first word	Yes
Poor mobility (trouble walking, cannot leave bed, falling)	Good comprehension	No
Swallowing problems (e.g. chokes, inhales food or drink, holds food in mouth)	Good comprehension	No
Skin breakdown (redness, skin tearing, pressure damage)	Several interviewees mostly associated the item with pressure ulcers and did not understand the initial translation for skin tearing	Yes
Difficulty communicating	Good comprehension	No
Sleeping problems	Good comprehension	No
Diarrhoea	Good comprehension	No
Hallucinations (seeing or hearing things not present) and/or delusions (fixed false beliefs)	Good comprehension, but the initial translation for “false” had a negative connotation	Yes
Agitation (restless, irritable, aggressive)	Good comprehension	No
Wandering (as a result of distress or putting person at risk)	Good comprehension without the explanation in parentheses	Yes
Has the person had any other symptoms? Please select one box to show how you feel each of these symptoms have affected the person over the past week (optional)	Good comprehension	No
Q3. Has s/he been feeling anxious or worried?	Some informants raised concerns about the first Swedish translation as referring only to everyday worrying and not to anxiety disorders	Yes
Q4. Have any of his/her family been anxious or worried about the person?	Good comprehension	No
Q5. Do you think s/he felt depressed?	Good comprehension	No
Q5b. Lost interest in things s/he would normally enjoy?	Good comprehension	No
Q6. Do you think s/he felt at peace?	Good comprehension	No
Q7. Has s/he been able to interact positively with others (e.g. staff, family, residents)?	Good comprehension	No
Q7b. Can s/he enjoy activities appropriate for his/her level of interests and abilities?	Good comprehension	No
Q8. Has his/her family had as much information as wanted?	Good comprehension	No
Q9. Have all practical problems been addressed? [e.g. hearing aids, foot care, glasses, diet]	The item was perceived as confusing regarding what problems to bring up there	Yes

Several questions asked about “the person”, which was found confusing regarding whom it referred to. This was changed to”the person with dementia” for clarity. Also, in the first version “his/her”, which appears several times in the instrument, was translated with the Swedish pronoun “hen”. The choice of “hen” was made as it can be used as a non-gender-specific pronoun. However, the interviews revealed that it could also be perceived as a trans-exclusive pronoun, thus excluding cis persons. Because of that, “hen” was removed and replaced with “the person with dementia”.

As noted in the translation process, the term “skin tearing” proved difficult to translate. The version of the Swedish IPOS-Dem used in the first interviews did not specify”hudfliksskada”. However, the phenomenon of skin tearing was not thought of as included in the broader wordings tested first. Several interviewees mostly associated the item with pressure ulcers and no other skin lesions. The Swedish term for skin tearing (“hudfliksskada”) identified in the earlier phases was tested separately during the interviews. The persons interviewed were not familiar with the term but understood its meaning when asked about the word. Thus, the term”hudfliksskada” was added to the item about skin breakdown. Also, the wording before the examples was changed from”for example” to”including” to further indicate that the examples were not the only imaginable skin lesions that could be included in the item.

*“You do not only associate to pressure [ulcers], it is also a bit more, a bit broader”* – participant number 5 (after”including” was added to the item).

In the item about hallucinations, the term “false” was changed from the Swedish “falska” to “felaktiga”, which was perceived as a more neutral expression.

Based on consistent feedback from the interviewees, “family” was changed from the literal translation “familj” to “närstående”, which corresponds roughly to “next of kin”. This can be seen as a cultural adaptation to the Swedish care context, in which the patient defines who is the nearest person(s) who should be informed and so on, regardless of whether they are blood relations. “Familj” in Swedish is a quite narrow term referring to spouses, children, siblings and/or parents. Many interviewees noted that for some patients/residents, the nearest person is a neighbour, a friend or a non-related person helping out with personal finances. This can be the case even if the person has children whom they are not very close to. The term “närstående” but not “familj” was broad enough to also include these persons.

*” There are people who do not have a family. Such a case is not very unusual in our ward. They may not have any [family]. Well, they may have a sibling who is 10 years older and they do not keep in touch. They are not married and do not have children. Maybe their closest person is a good friend or neighbour.”* – participant number 1.

The translation of “anxious or worried” used in the first interviews was “orolig eller ängslig”. These words were perceived by some interviewees as referring only to everyday worrying and not to anxiety disorders. It was even seen as diminishing to describe a person with an ongoing panic attack as “ängslig”. Instead, the term “ångestfylld” was chosen, which is a more medical term for anxious in Swedish.

The item about practical problems was perceived as confusing regarding what problems to bring up there. It was also not clear what to answer if the staff had tried to solve a problem but were waiting for someone else to handle it, for example, if the patient had been referred to a hearing aid department but had not yet received the hearing aid. There was also some confusion as to whether the item referred only to concerns that were the staff’s responsibility. Some interviewees referred to practical problems such as bill paying, which is not the responsibility for the staff in Sweden. To accommodate this problem and allow an easier follow-up process, a note was added which states that one can comment using free text.

After the sixth interview some minor changes were made to the IPOS-Dem based on recurring problems with wording in the interviews. The term “demenssjukdom” (dementia disease) was changed from just “dementia” on the front page for clarity. The words for drowsiness (dåsighet) and sleepiness (trötthet) were interchanged, because the latter was better understood and more commonly used. In the item about hallucination we added “for example” before “seeing or hearing things not present”, because several interviewees referred to hallucinations from other senses being relatively common. The item about sore or dry mouth was changed from “öm eller torr mun” to “munproblem inklusive smärta eller torrhet” (mouth problems including dryness or pain) because the first wording did not correspond to a common use of language in the Swedish context.

These changes were tested and perceived as well understood in the subsequent interviews. After changes were made to the item about skin damage, it was perceived as well understood.

### Usefulness

The interviewees found the tool interesting at their first impression and answered almost unanimously that the tool would be useful if implemented in standard care. It was perceived to include most clinically relevant symptoms, with the exception of urinary tract symptoms, and the included items were perceived as relevant. Possible hindrances were raised: the risk of time-consuming double documentation, how the tool could be implemented in digital medical records systems and whether it was overlapping with pre-existing assessment tools used for persons with dementia-related behavioural and psychological symptoms.

*“You try to have knowledge about [all included symptoms and problems] without this tool, but of course it gets a lot earlier if you have this and check off things.”* – participant number 7.

## Discussion

In this study, we describe the translation and cultural adaptation process of IPOS-Dem into Swedish. After the initial translation, the interviews identified several areas where clarification and adjustments to the Swedish care context were needed. The tool was perceived as clinically useful but, in some parts, overlapping with already implemented tools. The Swedish IPOS-Dem can now be used as a tool for assessing symptoms and problems for persons with advanced dementia. The Swedish translation of the instrument should be further examined for validity and reliability. This is to our knowledge the first holistic tool for assessing symptoms and problems in persons with advanced dementia with a validated Swedish translation.

As was also found in the study translating IPOS into Swedish [15], “overwhelmingly” could not be literally translated into Swedish. This led to the choice of using the same terms as in commonly used pain assessment tools [26, 27]. Several issues with the Swedish IPOS-Dem after the translation process were highlighted during the first interviews. An example is the term “wandering”, which is provided with an elucidation in the English version of the instrument – as a result of distress or putting the person at risk. Clarification of the term “wandering” was not considered to be necessary in the Swedish context. This illustrates the need for cultural adaptation, interviewing healthcare staff who will later use the tool.

This study highlights the importance of defining a distinct group of patients or residents with whom the tool will be used. Some interviewees associated the word “palliative” with end-of-life care. This term was later changed to “early integrated palliative”, which was recognised as referring to a wider population of persons with dementia.

Credibility, dependability, confirmability and transferability are often described as important for appraising the trustworthiness of the result. To achieve credibility, we asked the next respondent to reflect upon earlier respondents’ views. This was also a way of assessing the dependability of the result, and since we assessed that saturation was reached we conclude that the same result would be replicated if the study was done again. Transferability can be discussed. The result aims to be applicable in Sweden, it is however up to the user to put it in their own context.

Quality assessment and monitoring are important for healthcare development. Instruments can work as checklists and give structure to care provided by both experienced and inexperienced healthcare workers. A drawback is the risk of missing things that are not included in the instrument. Another conceivable drawback to implementing tools is the time-consuming process of documentation, especially if documentation of the instrument assessment is not incorporated into the modules for day-to-day documentation. A hindrance to implementation found in this study is that the tool is somewhat overlapping with pre-existing and already implemented tools. In this study we did not compare different instruments, which could be a focus in future studies. A possible approach for this in the future could also be to combine different elements to find the best possible tool for the Swedish care context.

### Strengths and limitations

This study conducted a translation and cultural adaptation of IPOS-Dem based on pre-existing guidelines for these steps: conceptual phase, forward translation, backward translation and interviews. However, the initial plan to conduct face-to-face interviews had to be rethought. When the Covid-19 pandemic was declared, three interviews had been conducted. To interview staff in dementia care during the pandemic meant that all subsequent interviews were conducted electronically. This implies a possible bias in the recruitment process. Some informants’ lack of familiarity with the use of digital tools may have been perceived as an obstacle to accepting the interview, and thereby, we may have introduced a selection bias in recruiting informants to our study. To compensate, additional interviews were conducted, even though we had assessed that the material was probably saturated after the fifth interview. The method to use managers to help recruit interview persons may have introduced a bias towards people with special interest in dementia care and palliative care. It is possible that other healthcare staff may not be as familiar with the terms used in the IPOS-Dem.

## Conclusions

The Swedish version of the IPOS-Dem can now be used as a tool for assessing palliative care related problems and concerns for persons with advanced dementia. Future studies can focus on implementation as well as examining validity and reliability of this tool in a Swedish context.

## Data Availability

The datasets generated and/or analysed during the current study are not publicly available due to privacy of respondents. Please contact the corresponding author for more information.

## Abbreviations

BPSD: behavioural and psychological symptoms of dementia
IPOS: Integrated Palliative care Outcome Scale
IPOS-Dem: Integrated Palliative care Outcome Scale for Dementia
POS: Palliative care Outcome Scale
